# The Effect of Intraoperative Hypotension on Postoperative Renal Function

**DOI:** 10.1007/s40140-023-00564-2

**Published:** 2023-06-14

**Authors:** Benjamin Kim, Gurleen Sangha, Amrik Singh, Christian Bohringer

**Affiliations:** 1 Department of Anesthesiology and Pain Medicine, University of California Davis Health, Sacramento, CA, USA; 2 Dayanand medical College and Hospital, Ludhiana, India

**Keywords:** Intraoperative hypotension, Acute kidney injury, Intravenous fluid management, Vasopressors, Goal-directed fluid therapy

## Abstract

**Purpose of Review:**

This review summarizes the most recent literature on the association between intraoperative hypotension (IOH) and the occurrence of postoperative acute kidney injury (AKI). It provides recommendations for the management of intraoperative blood pressure to reduce the incidence of postoperative AKI. Fluid management strategies, administration of vasopressor medications, and other methods for reducing the incidence of AKI are also briefly discussed.

**Recent Findings:**

Recent retrospective studies have demonstrated a solid association of IOH with postoperative AKI. IOH is associated not only with AKI but also with myocardial infarction, stroke, and death. Strict BP management to avoid a mean blood pressure less than 65mmHg is now recommended to reduce the incidence of postoperative AKI and other adverse outcomes.

**Summary:**

IOH is robustly associated with AKI, and intraoperative mean BP should be maintained above 65 mmHg at all times. The etiology of postoperative AKI is however multifactorial, and factors other than BP therefore also need to be considered to prevent it.

## Introduction

Postoperative acute kidney injury (AKI) is a common complication that is associated with the development of chronic kidney failure, prolonged hospitalization, higher cost of medical care, and increased mortality [1–6]. The incidence of AKI is variable and depends on pre-existing conditions as well as the nature of the surgical intervention. AKI occurs more often after emergency operations than after ambulatory procedures [7]. In a recent study in patients with fractured femur, the incidence of postoperative AKI was as high as 18% [8]. Evidence from retrospective multicenter trials indicates a solid association between intraoperative hypotension (IOH) and postoperative AKI [9, 10]. Correction of IOH has the potential to decrease postoperative AKI and the associated costs [11]. A recent multicenter retrospective cohort study in major non-cardiac procedures found not only an increased incidence of AKI but also elevated rates of myocardial infarction, stroke, and mortality in patients with IOH [12]. The same findings were made in a multicenter study in cardiac surgical patients [13]. The evidence implicating IOH in AKI is primarily derived from retrospective studies. It is an association and does not necessarily imply a causal relationship. Given the strong correlation of IOH with AKI and other poor outcomes in retrospective studies, however, it is unlikely that this topic will be studied in randomized prospective trials in the future because subjecting patients prospectively to IOH would be regarded as unethical

### Definition of IOH

Most studies defined IOH as a MAP less than 65 mmHg [14, 15]. Relative thresholds based on a drop from preoperative BP were found to be no more predictive for AKI than this absolute threshold of 65mmHg. There was also no clinically important association of preoperative BP with postoperative AKI. BP management under anesthesia is therefore now recommended by some to be based on intraoperative BP without regard to preoperative BP [14].

The autoregulatory curve is however shifted to the right in patients with significant preoperative hypertension [16, 17]. Patients with poorly controlled hypertension may therefore need to be treated with higher BP goals because aggressive treatment of hypertension in this group of patients is associated with an increase in the serum creatinine concentration [18]. In a multicenter study in septic ICU patients, there was a direct correlation of relative hypotension with AKI [19].

The Perioperative Quality Initiative consensus statement on intraoperative BP regarding the risks and outcomes for elective surgery also clearly highlighted that even brief durations of MAP less than 60–70mmHg are harmful during non-cardiac surgery [20]. However, there is significant variation in the definition of IOH in the literature [21]. Reported systolic BP thresholds vary from as low as 55mmHg [22] to as high as 110mmHg [23, 24]. When studies use a relative BP drop from preoperative levels instead of absolute values, there is a weaker association with detecting postoperative adverse events [21].

### Definition of AKI

AKI has historically been defined as a small elevation in serum creatinine, but new biomarkers are under investigation as rapidly obtainable and more accurate predictors of AKI [25]. Twenty-four-hour urine creatinine clearance is a more accurate test for AKI than a rise in serum creatinine, but it is difficult to obtain in routine clinical practice. A rise in serum creatinine from baseline has therefore been considered to be a reasonable substitute. Trying to standardize AKI using serum creatinine has however serious flaws because creatinine is affected by factors other than AKI like volume overload, nutrition, steroids, and muscle trauma. Several biomarkers for AKI like tissue inhibitor of metalloproteinase-2, insulin-like growth factor binding protein-7, and neutrophil gelatinase-associated lipocalin have the potential to substantially improve the diagnostic approach to AKI in the future [26, 27]. AKI is defined differently by different organizations. The Kidney Disease: Improving Global Outcomes (KDIGO) definition [28] differs from the Risk, Injury, Failure, Loss, and End-Stage Renal Disease (RIFLE), the AKI Network (AKIN), and the Society of Thoracic Surgeons (STS) criteria [29]. RIFLE uses Glomerular Filtration Rate (GFR) criteria in addition to serum creatinine and urine output criteria. GFR criteria were abandoned in the AKIN and KDIGO definitions. The Risk, Injury, and Failure categories of the RIFLE definition were replaced by AKIN and KDIGO stages 1, 2, and 3. In AKIN stage 1, an absolute rise in serum creatinine of more than 26.4 μmol/l was added to the relative increase of 150–200% in serum creatinine compared to baseline. This increase in serum creatinine of more than 26.4 μmol/l in AKIN stage 1 was replaced by an absolute rise in serum creatinine of more than 26.5 μmol/l in stage 1 of the KDIGO definition. Both in the RIFLE and KDIGO definitions, the increase in creatinine is defined to occur within 7 days, which contrasts with the 48 h used in the AKIN definition. In stage 3 of the AKIN and KDIGO definitions, the need for renal replacement therapy (RRT) was added. The categories Loss and End-Stage Kidney disease or equivalent (outcome) categories from the RIFLE definition were removed from the AKIN and KDIGO definitions. Urine output criteria are similar in RIFLE, AKIN, and KDIGO definitions.

AKIN and KDIGO detect more AKI patients than the RIFLE criteria, and this explains the large heterogeneity in the literature [30]. The current STS definition of acute renal failure is a threefold increase in serum creatinine, a creatinine greater than 4mg/dl, or the initiation of dialysis. This definition fails to identify the vast majority of patients with AKI [31].

### Risk Factors for AKI

Independent risk factors for perioperative AKI include advanced age, pre-existing hypertension, active congestive heart failure, chronic kidney disease, insulin-dependent diabetes mellitus, peripheral vascular disease, the presence of ascites, high body mass index, pulmonary disease, and the African American race (Table 1) [32]. Patients with sepsis and systemic inflammatory response syndrome are also at increased risk, and it appears that there is a microvascular component involved in in the development of AKI [33]. Venous congestion in patients with right ventricular dysfunction is another risk factor for AKI. While increased intravascular volume is usually protective against AKI in patients with venous congestion from congestive heart failure, it is associated with an increased risk of AKI [34]. Procedures associated with AKI are cardiac, thoracic, orthopedic, vascular, urologic, and ear, nose, and throat operations. A prediction model has been developed and validated for postoperative AKI after non-cardiac surgery [35]. Prevention of IOH in these particularly at-risk patient groups and surgical interventions is therefore very important. Serum creatinine should also be measured postoperatively in these at-risk patients because perioperative AKI often goes unrecognized [36].

### Measurement of BP

Outcome studies looking at the association of IOH and AKI usually measure mean arterial pressure (MAP). This is the most reliable method of identifying true IOH because systolic and diastolic pressures are more susceptible to measurement errors resulting from wave summation artifacts. The MAP is the most robust measurement and should be used to guide the management of intraoperative BP [37]. BP cuffs need to be of the correct size for the patient to be able to accurately identify IOH. A cuff that is too large will underestimate BP, and a cuff that is too small will overestimate BP. Measurement errors are especially common in obese patients with large arms when standard cylindrical cuffs are used [38]. Newer non-invasive continuous BP monitors have become available that measure BP in the digital arteries via a finger cuff. A recent study in vascular surgical patients found the use of ClearSight is a viable alternative to invasive BP measurement via a radial arterial line [39].

## Methods to Avoid Hypotension

### Optimizing Preload and Intravascular Volume Status

Adequate hydration and maintaining optimal preload to the left ventricle are crucial for maintaining organ perfusion. Fluid management should be individualized and optimized for every single patient [40]. Dehydration has been clearly implicated in the pathophysiology of postoperative AKI [41, 42]. Preoperative volume status is unfortunately not always given sufficient attention in clinical practice and should be thoroughly evaluated especially prior to emergency operations. A recent multicenter prospective randomized trial showed a higher rate of AKI when restrictive fluid management was used in patients at risk for complications during major abdominal surgery [43]. Consideration also needs to be given to maintaining the normal composition of the circulating blood. Clinicians need to understand the Starling forces that affect the movement of fluid across a semipermeable membrane like the glomerulus. Anesthesiologists should not extrapolate results obtained from studies in intensive care patients with sepsis to their patients with significant hemorrhage in the operating room. The pathophysiological origin of the hypotension in these two groups of patients is very different. It is therefore not surprising that the same therapeutic intervention has a different outcome in both of these different clinical scenarios.

### Vasopressor Agent Use

Most drugs used to induce general anesthesia cause some degree of vasodilation. Dilation of the venous system results in reduced left ventricle preload and then reflected in reduced MAP and cardiac output. Administration of vasopressors like phenylephrine has been shown to increase rather than decrease cardiac output by raising cardiac preload back to normal levels in patients with anesthesia-induced hypotension [44]. While vasopressors can improve perfusion in hypotension caused by vasodilation from anesthetic drugs, they should however not be used to treat hypotension due to dehydration or hypovolemia [45].

### Balance Between Intravenous Fluid and Vasopressors

Keeping MAP above 65 mmHg does not necessarily guarantee sufficient organ perfusion and maintenance tissue oxygen delivery. Clinicians should not automatically assume that organ perfusion is adequate simply because BP goals are met. When managing BP intraoperatively, an appropriate balance needs to be maintained between vasopressor medication and intravenous fluids to ensure that perfusion remains acceptable. Clinical signs of peripheral perfusion like skin color, capillary refill time, temperature of the extremities, and urine output need to be assessed continuously throughout the procedure. Skin pallor, slow refill time, and poor urine output should be treated with intravenous fluid rather than vasopressor medications. In patients with poor ejection fraction, a beta-1 agonist may need to be used in combination with intravenous fluids. The patient needs to be assessed for clinical signs of sympathetic nervous system activation like tachycardia, narrow pulse pressure, pallor, diaphoresis, and mydriasis throughout the procedure because these findings can be indicators of significant hypovolemia. Positioning changes may also profoundly affect venous return and cardiac output [46]. Steep reverse Trendelenburg position may result in IOH due to decreased venous return which may need to be treated with vasopressor medications.

The notion that maintaining MAP above 65 mmHg without considering indictors of cardiac output may not be sufficient was confirmed in a recent prospective trial in major non-cardiac surgery that failed to show a reduction in AKI despite a 60% reduction in hypotensive time with a MAP less than 65 mmHg [47]. The patients in the group with a mean BP target of greater than 75mmHg received significantly higher cumulative doses of vasopressors. Cardiac output was not measured in this study, and excessive use of vasopressor medications instead of intravenous fluid in the high BP group could explain this unexpected finding.

Careful clinical assessment therefore needs to be employed when administering vasopressors. In a recent retrospective cohort study in elderly patients undergoing non-cardiac surgery, high vasopressor use was associated with postoperative AKI, independent of hypotension identified from anesthetic records [48]. A high vasopressor dose in this study was defined as a cumulative dose of greater than 20 mg of metaraminol, norepinephrine, or phenylephrine or greater than 10mg epinephrine. Every 5mg increase of the total dose of vasopressors used during the surgery was associated with an 11% increase in the odds of AKI.

### Goal-Directed Fluid Therapy

Goal-directed fluid therapy utilizing dynamic indices of intravascular fluid volume like stroke volume variation have become widely used in high-risk patients. Measurements derived from this pulse contour analysis-based technology have been well validated when compared to values obtained from a pulmonary artery catheter [49]. A systematic review of goal-directed fluid therapy on renal function in critically ill patients concluded that this may be an effective method to prevent AKI in critically ill patients [50]. A prospective, randomized, controlled, and blinded study in patients undergoing major elective non-cardiac surgery failed however to demonstrate a benefit of goal-directed therapy in the prevention of AKI [51]. The detailed physiologic data analyzed in this study did nevertheless confirm the expert consensus that even relatively short periods of IOH requiring vasopressors contribute to perioperative AKI. A recent trial in open radical cystectomy patients unexpectedly found an even higher rate of AKI in the goal-directed fluid management group than in the standard fluid management group [52]. The goal-directed group had lower urine output and lower intraoperative and 6-h recovery room fluid intake. The reduced fluid given to the goal-directed group may have caused the increased rate of AKI. Failure of this new strategy to consistently prevent AKI in the perioperative setting may be due to insufficient understanding of the factors that affect stroke volume variation. Increased airway pressure due to positive pressure ventilation, increased positive end expiratory pressure, bronchospasm, and raised intra-abdominal pressure increase stroke volume variation [53–55]. Hypovolemia can therefore be present despite an apparently normal stroke variation. Intravenous fluids may consequently need to be administered on occasion despite a normal stroke volume variation to prevent AKI.

## Conclusions

Retrospective observational studies show strong association between IOH and postoperative AKI. Even though this association does not prove that IOH is the cause of AKI, the current evidence strongly suggests that intraoperative MAP should be maintained above 65 mmHg at all times. IOH has also been associated with postop myocardial infarction, stroke, and death. Prospective studies with treatment arms randomizing patients to be deliberately exposed to IOH are unlikely to be conducted in the future because this would be regarded as unethical.

## Figures and Tables

**Table 1:** Risk factors for perioperative AKI

Advanced age
Pre-existing hypertension
Congestive heart failure
Chronic kidney disease
Insulin-dependent diabetes
Peripheral vascular disease
Ascites
High body mass index

*AKI* acute kidney injury

## References

[1] Hobson C, Ozrazgat-Baslanti T, Kuxhausen A, Thottakkara P, Efron PA, Moore FA, Moldawer LL, Segal MS, Bihorac A. Cost and mortality associated with postoperative acute kidney injury. Ann Surg. 2015;261:1207–14. 10.1097/SLA.0000000000000732. 24887982 PMC4247993

[2] Biteker M, Dayan A, Tekkeşin Aİ, Can MM, Taycı İ, İlhan E, Şahin G. Incidence, risk factors, and outcomes of perioperative acute kidney injury in noncardiac and nonvascular surgery. Am J Surg. 2014;207:53–9. 10.1016/j.amjsurg.2013.04.006. 24050540

[3] Kork F, Balzer F, Spies CD, Wernecke KD, Ginde AA, Jankowski J, Eltzschig HK. Minor postoperative increases of creatinine are associated with higher mortality and longer hospital length of stay in surgical patients. Anesthesiology. 2015;123:1301–11. 10.1097/ALN.0000000000000891. 26492475 PMC4679549

[4] O’Connor ME, Hewson RW, Kirwan CJ, Ackland GL, Pearse RM, Prowle JR. Acute kidney injury and mortality 1 year after major non-cardiac surgery. Br J Surg. 2017;104:868–76. 10.1002/bjs.10498. 28218392

[5] Joosten A, Lucidi V, Ickx B, Van Obbergh L, Germanova D, Berna A, Alexander B, Desebbe O, Carrier FM, Cherqui D, Adam R, Duranteau J, Saugel B, Vincent JL, Rinehart J, Van der Linden P. Intraoperative hypotension during liver transplant surgery is associated with postoperative acute kidney injury: a historical cohort study. BMC Anesthesiol. 2021;21:12. 10.1186/s12871-020-01228-y. 33430770 PMC7798188

[6] Privratsky JR, Krishnamoorthy V, Raghunathan K, Ohnuma T, Rasouli MR, Long TE, Sigurdsson MI. Postoperative acute kidney injury is associated with progression of chronic kidney disease independent of severity. Anesth Analg. 2022;134:49–58. 10.1213/ANE.0000000000005702. 34908546

[7] Prowle JR, Forni LG, Bell M, Chew MS, Edwards M, Grams ME, Grocott MP, Liu KD, Mcilroy D, Murray PT, Ostermann M. Postoperative acute kidney injury in adult non-cardiac surgery: joint consensus report of the Acute Disease Quality Initiative and PeriOperative Quality Initiative. Nat Rev Nephrol. 2021;17:605–18. 10.1038/s41581-021-00418-2. 33976395 PMC8367817

[8] Jang WY, Jung JK, Lee DK, Han SB. Intraoperative hypotension is a risk factor for postoperative acute kidney injury after femoral neck fracture surgery: a retrospective study. BMC Musculoskelet Disord. 2019;20:131. 10.1186/s12891-019-2496-1. 30917804 PMC6438026

[9] Mathis MR, Naik BI, Freundlich RE, Shanks AM, Heung M, Kim M, Multicenter perioperative outcomes group investigators. Preoperative risk and the association between hypotension and postoperative acute kidney injury. Anesthesiology. 2020;(132):461–75. 10.1097/ALN.0000000000003063. Erratum in: Anesthesiology. 2020 Jan 6 PMC7015776 31794513

[10] Wesselink EM, Kappen TH, Torn HM, Slooter AJC, van Klei WA. Intraoperative hypotension and the risk of postoperative adverse outcomes: a systematic review. Br J Anaesth. 2018;121:706–21. 10.1016/j.bja.2018.04.036. 30236233

[11] Shaw AD, Khanna AK, Smischney NJ, Shenoy AV, Boero IJ, Bershad M, Hwang S, Chen Q, Stapelfeldt WH. Intraoperative hypotension is associated with persistent acute kidney disease after noncardiac surgery: a multicentre cohort study. Br J Anaesth. 2022;129:13–21. 10.1016/j.bja.2022.03.027. 35595549

[12] Sessler DI, Bloomstone JA, Aronson S, Berry C, Gan TJ, Kellum JA, Perioperative quality Initiative consensus statement on intraoperative blood pressure, risk and outcomes for elective surgery. Br J Anaesth. 2019;122:563–74. 10.1016/j.bja.2019.01.013. 30916004

[13] Sun LY, Wijeysundera DN, Tait GA, Beattie WS. Association of intraoperative hypotension with acute kidney injury after elective noncardiac surgery. Anesthesiology. 2015;123:515–23. 10.1097/ALN.0000000000000765. 26181335

[14] Salmasi V, Maheshwari K, Yang D, Mascha EJ, Singh A, Sessler DI, Kurz A. Relationship between intraoperative hypotension, defined by either reduction from baseline or absolute thresholds, and acute kidney and myocardial injury after noncardiac surgery: a retrospective cohort analysis. Anesthesiology. 2017;126:47–65. 10.1097/ALN.0000000000001432. 27792044

[15] Gregory A, Stapelfeldt WH, Khanna AK, Smischney NJ, Boero IJ, Chen Q, Stevens M, Shaw AD. Intraoperative hypotension is associated with adverse clinical outcomes after noncardiac surgery. Anesth Analg. 2021;132:1654–65. 10.1213/ANE.0000000000005250. 33177322 PMC8115733

[16] Carlström M, Wilcox CS, Arendshorst WJ. Renal autoregulation in health and disease. Physiol Rev. 2015;95(2):405–511. 10.1152/physrev.00042.2012. 25834230 PMC4551215

[17] Suarez J, Busse LW. New strategies to optimize renal haemodynamics. Curr Opin Crit Care. 2020;26(6):536–42. 10.1097/MCC.0000000000000774. 33044238

[18] Palmer BF. Impaired renal autoregulation: implications for the genesis of hypertension and hypertension-induced renal injury. Am J Med Sci. 2001;321(6):388–400. 10.1097/00000441-200106000-00005. 11417753

[19] Panwar R, Tarvade S, Lanyon N, Saxena M, Bush D, Hardie M, Attia J, Bellomo R, Van Haren F, REACT Shock Study Investigators and Research Coordinators. Relative hypotension and adverse kidney-related outcomes among critically ill patients with shock. A multicenter, prospective cohort study. Am J Respir Crit Care Med. 2020;202(10):1407–18. 10.1164/rccm.201912-2316OC. 32614244

[20] de la Hoz MA, Rangasamy V, Bastos AB, Xu X, Novack V, Saugel B, Subramaniam B. Intraoperative hypotension and acute kidney injury, stroke, and mortality during and outside cardiopulmonary bypass: a retrospective observational cohort study. Anesthesiology. 2022;136:927–39. 10.1097/ALN.0000000000004175. 35188970

[21] Weinberg L, Li SY, Louis M, Karp J, Poci N, Carp BS, Miles LF, Tully P, Hahn R, Karalapillai D, Lee DK. Reported definitions of intraoperative hypotension in adults undergoing non-cardiac surgery under general anaesthesia: a review. BMC Anesthesiol. 2022;22:69. 10.1186/s12871-022-01605-9. 35277122 PMC8915500

[22] Miyazaki R, Kajiyama E, Kandabashi T, Hoka S. An evaluation of perioperative acute kidney injury during laparoscopic sleeve gastrectomy using the acute kidney injury network classification. Anesthesia Resuscitation. 2016;52:51–5.

[23] Sheffy N, Bentov I, Mills B, Nair BG, Rooke GA, Vavilala MS. Perioperative hypotension and discharge outcomes in non-critically injured trauma patients, a single Centre retrospective cohort study. Injury. 2017;48:1956–63. 10.1016/j.injury.2017.06.023. 28733043

[24] Palmer A, Taitsman LA, Reed MJ, Nair BG, Bentov I. Utility of geriatric assessment in the projection of early mortality following hip fracture in the elderly patients. Geriatr Orthop Surg Rehabil. 2018;9:2151459318813976. 10.1177/2151459318813976. PMC6287303 30546923

[25] Goren O, Matot I. Perioperative acute kidney injury. Br J Anaesth. 2015;115:ii3–14. 10.1093/bja/aev380. 26658199

[26] Ronco C, Bellomo R, Kellum JA. Acute kidney injury. Lancet. 2019;394(10212):1949–64. 10.1016/S0140-6736(19)32563-2. 31777389

[27] Cummings JJ, Shaw AD, Shi J, Lopez MG, O’Neal JB, Billings FT 4th. Intraoperative prediction of cardiac surgery-associated acute kidney injury using urinary biomarkers of cell cycle arrest. J Thorac Cardiovasc Surg. 2019;157:1545–53. 10.1016/j.jtcvs.2018.08.090. 30389130 PMC6431272

[28] Rovin BH, Adler SG, Barratt J, Bridoux F, Burdge KA, Chan TM, Cook HT, Fervenza FC, Gibson KL, Glassock RJ, Jayne DR. Executive summary of the KDIGO 2021 Guideline for the management of glomerular diseases. Kidney Int. 2021;100:753–79. 10.1016/j.kint.2021.05.015. 34556300

[29] Thomas ME, Blaine C, Dawnay A, Devonald MA, Ftouh S, Laing C, Latchem S, Lewington A, Milford DV, Ostermann M. The definition of acute kidney injury and its use in practice. Kidney Int. 2015;87:62–73. 10.1038/ki.2014.328. 25317932

[30] Koeze J, Keus F, Dieperink W, van der Horst IC, Zijlstra JG, van Meurs M. Incidence, timing and outcome of AKI in critically ill patients varies with the definition used and the addition of urine output criteria. BMC Nephrol. 2017;18:70. 10.1186/s12882-017-0487-8. 28219327 PMC5319106

[31] Engelman DT, Schwann TA. Commentary: a little is way too much: what we have learned about perioperative acute kidney injury. J Thorac Cardiovasc Surg. 2021;162(1):153–4. 10.1016/j.jtcvs.2019.12.100. 32061391

[32] Gumbert SD, Kork F, Jackson ML, Vanga N, Ghebremichael SJ, Wang CY, Eltzschig HK. Perioperative acute kidney injury. Anesthesiology. 2020;132:180–204. 10.1097/ALN.0000000000002968. 31687986 PMC10924686

[33] Zafrani L, Ince C. Microcirculation in acute and chronic kidney diseases. Am J Kidney Dis. 2015;66:1083–94. 10.1053/j.ajkd.2015.06.019. 26231789

[34] Lopez MG, Shotwell MS, Morse J, Liang Y, Wanderer JP, Absi TS, Balsara KR, Levack MM, Shah AS, Hernandez A, Billings FT 4th. Intraoperative venous congestion and acute kidney injury in cardiac surgery: an observational cohort study. Br J Anaesth. 2021;126(3):599–607. 10.1016/j.bja.2020.12.028. 33549321 PMC8014941

[35] Park S, Cho H, Park S, Lee S, Kim K, Yoon HJ, Park J, Choi Y, Lee S, Kim JH, Kim S, Chin HJ, Kim DK, Joo KW, Kim YS, Lee H. Simple postoperative AKI risk (SPARK) classification before noncardiac surgery: a prediction index development study with external validation. J Am Soc Nephrol. 2019;30:170–81. 10.1681/ASN.2018070757. 30563915 PMC6317608

[36] Meersch M, Schmidt C, Zarbock A. Perioperative acute kidney injury: an under-recognized problem. Anesth Analg. 2017;125:1223–32. 10.1213/ANE.0000000000002369. 28787339

[37] Lam S, Liu H, Jian Z, Settels J, Bohringer C. Intraoperative invasive blood pressure monitoring and the potential pitfalls of invasively measured systolic blood pressure. Cureus. 2021;13(8):e17610. 10.7759/cureus.17610. 34646661 PMC8483407

[38] Palatini P, Benetti E, Fania C, Ermolao A, Spinella P, Battista F, Gasperetti A, Saladini F. Effect of the shape of the cuff on blood pressure measurement in people with large arms. Blood Press. 2020;29:241–6. 10.1080/08037051.2020.1738913. 32172593

[39] Tanioku T, Yoshida A, Aratani Y, Fujii K, Kawamata T. Validation of noninvasive continuous arterial pressure measurement by ClearSight System^™^ during induction of anesthesia for cardiovascular surgery. BMC Anesthesiol. 2020;20:176. 10.1186/s12871-020-01091-x. 32690040 PMC7370521

[40] De Backer D, Aissaoui N, Cecconi M, Chew MS, Denault A, Hajjar L, Hernandez G, Messina A, Myatra SN, Ostermann M, Pinsky MR, Teboul JL, Vignon P, Vincent JL, Monnet X. How can assessing hemodynamics help to assess volume status? Intensive Care Med. 2022;48:1482–94. 10.1007/s00134-022-06808-9. 35945344 PMC9363272

[41] Scholz H, Boivin FJ, Schmidt-Ott KM, Bachmann S, Eckardt KU, Scholl UI, Persson PB. Kidney physiology and susceptibility to acute kidney injury: implications for renoprotection. Nat Rev Nephrol. 2021;17:335–49. 10.1038/s41581-021-00394-7. 33547418

[42] Canet E, Bellomo R. Perioperative renal protection. Curr Opin Crit Care. 2018;24:568–74. 10.1097/MCC.0000000000000560. 30308540

[43] Ripollés-Melchor J, Aldecoa C, Alday-Muñoz E, Del Río S, Batalla A, Del-Cojo-Peces E, Uña-Orejón R, Muñoz-Rodés JL, Lorente JV, Espinosa ÁV, Ferrando-Ortolà C, Jover JL, Abad-Gurumeta A, Ramírez-Rodríguez JM. Abad-Motos A; investigators’ group of the POWER and POWER 2 studies for the Spanish Perioperative Audit, Research Network (RedGERM-SPARN). Intraoperative crystalloid utilization variability and association with postoperative outcomes: a post hoc analysis of two multicenter prospective cohort studies. Rev Esp Anestesiol Reanim (Engl Ed). 2021;68:373–83. 10.1016/j.redare.2021.07.004. 34364826

[44] Kalmar AF, Allaert S, Pletinckx P, Maes JW, Heerman J, Vos JJ, Struys MMRF, Scheeren TWL. Phenylephrine increases cardiac output by raising cardiac preload in patients with anesthesia induced hypotension. J Clin Monit Comput. 2018;32:969–76. 10.1007/s10877-018-0126-3. 29569112 PMC6209056

[45] Myles PS, Bellomo R, Corcoran T, Forbes A, Peyton P, Story D, Christophi C, Leslie K, McGuinness S, Parke R, Serpell J, Chan MTV, Painter T, McCluskey S, Minto G, Wallace S; Australian and New Zealand College of Anaesthetists Clinical Trials Network and the Australian and New Zealand Intensive Care Society Clinical Trials Group. Restrictive versus liberal fluid therapy for major abdominal surgery. N Engl J Med. 2018:378:2263–2274. 10.1056/NEJMoa1801601. 29742967

[46] Tansey EA, Montgomery LEA, Quinn JG, Roe SM, Johnson CD. Understanding basic vein physiology and venous blood pressure through simple physical assessments. Adv Physiol Educ. 2019;43:423–9. 10.1152/advan.00182.2018. 31408386

[47] Wanner PM, Wulff DU, Djurdjevic M, Korte W, Schnider TW, Filipovic M. Targeting higher intraoperative blood pressures does not reduce adverse cardiovascular events following noncardiac surgery. J Am Coll Cardiol. 2021;78:1753–64. 10.1016/j.jacc.2021.08.048. 34711333

[48] Ariyarathna D, Bhonsle A, Nim J, Huang CKL, Wong GH, Sim N, Hong J, Nan K, Lim AKH. Intraoperative vasopressor use and early postoperative acute kidney injury in elderly patients undergoing elective noncardiac surgery. Ren Fail. 2022;44:648–59. 10.1080/0886022X.2022.2061997. 35403562 PMC9009951

[49] Kobe J, Mishra N, Arya VK, Al-Moustadi W, Nates W, Kumar B. Cardiac output monitoring: technology and choice. Ann Card Anaesth. 2019;22:6–17. 10.4103/aca.ACA_41_18. 30648673 PMC6350438

[50] Zhao CC, Ye Y, Li ZQ, Wu XH, Zhao C, Hu ZJ. Effect of goal-directed fluid therapy on renal function in critically ill patients: a systematic review and meta-analysis. Ren Fail. 2022;44:777–89. 10.1080/0886022X.2022.2072338. 35535511 PMC9103701

[51] Patel A, Prowle JR, Ackland GL, Investigators POM-OS. Postoperative goal-directed therapy and development of acute kidney injury following major elective noncardiac surgery: post-hoc analysis of POM-O randomized controlled trial. Clin Kidney J. 2017;10:348–56. 10.1093/ckj/sfw118. 28616213 PMC5466093

[52] Arslan-Carlon V, Tan KS, Dalbagni G, Pedoto AC, Herr HW, Bochner BH, Cha EK, Donahue TF, Fischer M, Donat SM. Goal-directed versus standard fluid therapy to decrease ileus after open radical cystectomy: a prospective randomized controlled trial. Anesthesiology. 2020;133:293–303. 10.1097/ALN.0000000000003367. 32472804 PMC8320377

[53] Kang WS, Kim JY, Woo NS, Yoon TG. The influence of different mechanical ventilator settings of peak inspiratory pressure on stroke volume variation in pediatric cardiac surgery patients. Korean J Anesthesiol. 2014 May;66(5):358–63. 10.4097/kjae.2014.66.5.358. 24910727 PMC4041954

[54] Kawazoe Y, Nakashima T, Iseri T, Yonetani C, Ueda K, Fujimoto Y, Kato S. The impact of inspiratory pressure on stroke volume variation and the evaluation of indexing stroke volume variation to inspiratory pressure under various preload conditions in experimental animals. J Anesth. 2015;29(4):515–21. 10.1007/s00540-015-1995-y. 25771761 PMC4543412

[55] Jacques D, Bendjelid K, Duperret S, Colling J, Piriou V, Viale JP. Pulse pressure variation and stroke volume variation during increased intra-abdominal pressure: an experimental study. Crit Care. 2011;15(1):R33. 10.1186/cc9980. 21247472 PMC3222069

